# Molecular detection of *Oxyspirura* larvae in arthropod intermediate hosts

**DOI:** 10.1007/s00436-018-5756-3

**Published:** 2018-01-25

**Authors:** Sadia Almas, Anna G. Gibson, Steven M. Presley

**Affiliations:** The Institute of Environmental and Human Health, Texas Tech University, Box 41163, Lubbock, TX 79409-1163 USA

**Keywords:** *Oxyspirura petrowi*, Intermediate hosts, Galliformes, Parasitic nematodes, Bobwhite quail, Grasshopper, Cockroach

## Abstract

To determine potential intermediate hosts of *Oxyspirura petrowi*, a common nematode eyeworm of wild gallinaceous birds, various arthropod species including red harvester ants, beetles, wood cockroaches, crickets, grasshoppers, katydids, and desert termites were screened for the presence of *O. petrowi* using specific polymerase chain reaction (PCR) primers targeting the internal transcribed spacer 2 region (ITS2) of the eyeworm ribosomal deoxyribonucleic acid (rDNA). This is the first study to investigate the intermediate hosts of *O. petrowi* utilizing molecular techniques. We determined 38% (13/34) of the cockroaches, 27% (3/11) of the crickets, and 23% (68/289) of the grasshoppers which were positive for *O. petrowi*. Identifying potential intermediate hosts of *O. petrowi* is essential to better understanding the epizoology of the eyeworm’s transmission mechanics and to controlling infections in wild gallinaceous birds.

## Introduction

**Fig. 1:**
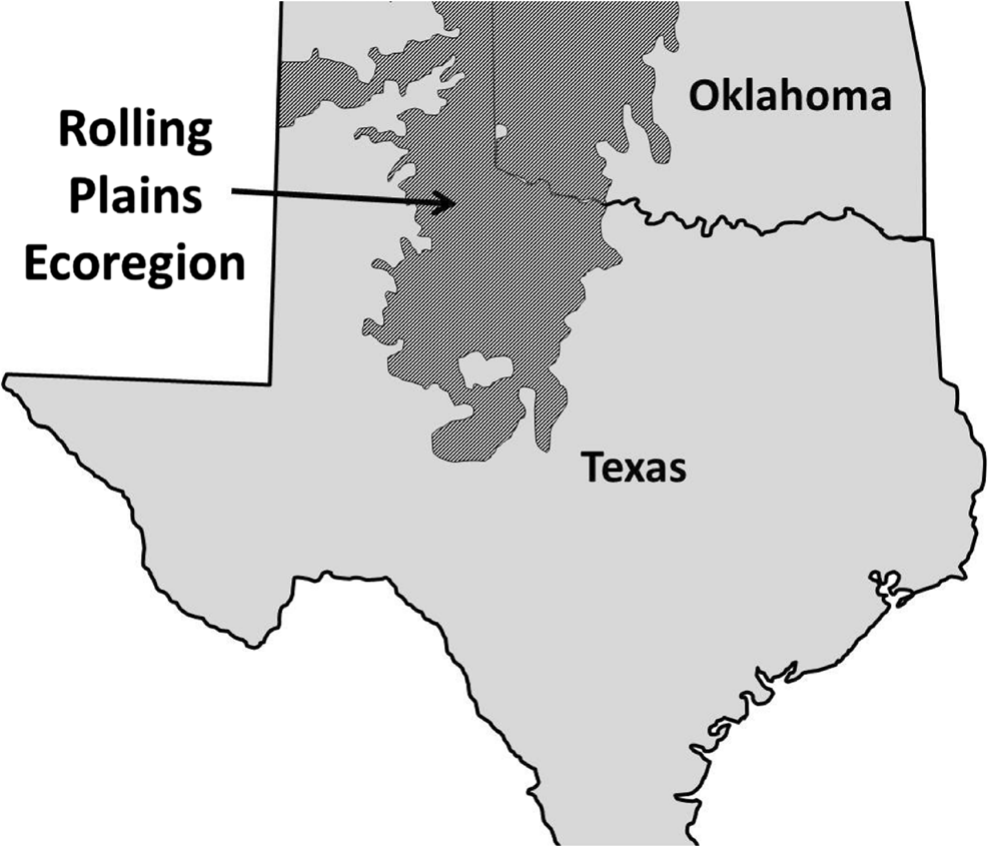
Map of the geographic area designated as the Rolling Plains ecoregion of Texas and Oklahoma, USA.

The long-term, and recently (2011–2013) more rapid, decline of northern bobwhite (*Colinus virginianus*) numbers in the Rolling Plains ecoregion of Texas and Oklahoma was investigated to determine factors that may be influencing their abundance (Fig. 1). During this study, 348 northern bobwhites were screened for different pathogens. Infection rates of 41.4% of bobwhite collected from individual sites in the Rolling Plains ecoregion by the parasitic nematode eyeworm *Oxyspirura petrowi* (Spirurida:Thelaziidae) was reported (Dunham et al. 2016).

Typically, these eyeworms are observed on the corneal surface under the nictitating membrane, in nasolacrimal ducts and in conjunctival sacs, causing cellular damage to these tissues; however, their effect on the survivability of their hosts is unknown. (Rodriguez-Tovar et al. 2008; Bruno et al. 2015). Previously, Landgrebe et al. 2007 reported an *O. petrowi* prevalence of 56% in scaled quail (*Callipepla squamata*) surveyed in Texas (Landgrebe et al. 2007). *Oxyspirura petrowi* is widely geographically distributed and occurs in a wide range of definitive hosts. *Oxyspirura petrowi* commonly infects wild birds of the order Galliformes in North America and was first reported in Michigan, USA in 1931, and has been reported infecting ruffed grouse (*Bonasa umbellus*), greater prairie chicken (*Tympanuchus cupido*), ring-necked pheasant (*Phasianus colchicus*), and sharp-tailed grouse *(Tympanuchus phasianellus*) (Cram 1937; Mcclure 1949; Pence 1972; Pence 1975; Pence et al. 1980). *Oxyspirura* spp*.* are spiruroid nematodes and include approximately 70 known species, three of which (*O. mansoni, O. petrowi*, and *O. pusillae*) have been reported in North America (Pence and Sell 1979). Typically these eyeworms are observed on the corneal surface under the nictitating membrane, in nasolacrimal ducts, and in conjunctival sacs, causing cellular damage to these tissues (Rodriguez-Tovar et al. 2008; Bruno et al. 2015).

The life cycle of *O. petrowi* is not well described, but it is assumed to be similar to that of *O. mansoni*, a common eyeworm of poultry. *Oxyspirura mansoni* requires an intermediate host such as a cockroach (Blattodea) to facilitate transmission to its definitive host. The eyeworm life cycle involves 50–100 days of stadial progression in the intermediate host, and 48 days in the definitive host (e.g., chicken) (Mcclure 1949). The importance of the intermediate hosts in the life cycle of *O. mansoni* was confirmed after futile attempts to directly infect chickens one to another using embryonated nematode eggs or first-stage eyeworm larvae (Cram 1937). Observational reports suggest various insects such as flies and grasshoppers may serve as intermediate hosts for *O. petrowi* (Mcclure 1949).

The high prevalence of *O. petrowi* reported in bobwhite and other wild game birds, and the limited knowledge of its transmission mechanisms, necessitate the development of a sensitive, specific, and reliable method to screen different arthropods for the presence of eyeworms. Typically, detection of nematodes in insects involves dissecting the insect and recovering nematodes, then identifying different specimens based on morphological characteristics. This process can be time consuming and its accuracy is highly dependent on the experience of the investigator, as it may be difficult to distinguish between different species of nematodes, especially the larval stages (Perera et al. 2013). We report here the development of a polymerase chain reaction (PCR)-based technique for the detection of any *O. petrowi* life stages in arthropods, for the purpose of learning more about its potential intermediate hosts. This is the first study to identify a range of potential arthropod intermediate hosts of *O. petrowi* based on molecular techniques. Due to their rapid evolution, high variation in their length and nucleotide contents between closely related species, the internal transcribed spacer (ITS) region of the ribosomal nucleic acid (rRNA) gene was chosen as target for developing species-specific primers.

## Materials and methods

This study was conducted in accordance with ethical standards and approved animal care and use protocols (Texas Tech University, Institutional Animal Care and Use Committee protocol No. 11049–070). Eyeworms were collected from the eyes of euthanized bobwhite and stored in 70% ethanol, then identified initially on the basis of morphological characteristics using a Nikon microscope (TE 2000 U) at 400× and 1000× magnification, after which they were submitted to the U.S. National Parasite Collection for confirmation (USNPC 106874). Deoxyribonucleic acid (DNA) was extracted from single nematode using the DNeasy Blood & Tissue Kit (QIAGEN, Maryland, USA) according to the manufacturer’s instructions. The internal transcribed spacer (ITS) region was amplified using the forward primer SPIR 18 and the reverse primer 28S/408R/20 (Ivanova et al. 2007; Makouloutou et al. 2013).The PCR product, ~ 1200 base pair (bp), was excised and cloned into JM109 competent cells (Promega Corp., Wisconsin, USA.) according to the manufacturer’s instructions. Plasmids were extracted and submitted for genetic sequencing to the University of Maine DNA Sequencing Facility (Orono, Maine, USA). Based on the sequence obtained, the primers OP_3_, 5′ TGTTGTGGAGCAGTTAAAATCC-3′, and OP_4_, 5′AACGTTATTGTTGCCATATGCT-3′, were designed to amplify a 197-bp region of ITS2.

To ensure specificity, the intraspecific variation in the ITS region of this genus was investigated. The sequence of the ITS1 region of *O. petrowi* was aligned with the corresponding region of *O. conjunctivalis*, which was the only *Oxyspirura* spp. with a sequence available in the National Center for Biotechnology Information (NCBI) database (accession number EF417873). Primers were synthesized by Integrated DNA Technologies, Inc. (IDT) (Iowa, USA). PCR conditions included a 2-minute denaturation step at 94 °C, followed by 35 cycles of 94 °C for 30 s, 55 °C for 30 s, and 72 °C for 1 min, with a final elongation step at 72 °C for 10 min. The specificity of primers was verified using the Basic Local Alignment Search Tool (BLAST) against the NCBI database. To further confirm the specificity of the primers, DNA isolated from the nematode *Strongyloides stercoralis* was used as a negative control. To determine the sensitivity of the primers, PCR was performed with 100 ng–1 pg of template DNA from *O. petrowi*.

Arthropod specimens were collected during biannual trapping of quail during the months of August and October (2012 and 2013) from several ranches across the Rolling Plains ecoregion of Texas and western Oklahoma. Various insects, including red harvester ants (Order: Hymenoptera), beetles (Order: Coleoptera), wood cockroaches (Order: Blattodea), crickets (Order: Orthoptera), grasshoppers (Order: Orthoptera), katydids (Order: Orthoptera), and desert termites (Order: Isoptera) were collected in close proximity to bobwhite quail trapping sites using baited petri plates, insect sweep nets, commercially available pheromone traps, and bread- and beer-baited traps as described by Pechal and others (Pechal et al. 2007). Collected specimens were preserved in 70% ethanol and stored at − 20 °C until analysis. Arthropods were identified to the lowest taxonomic level possible using morphological keys (Capinera et al. 2005) (Arnet 2000). Arthropods that could not be identified based on morphological characteristics were identified by direct sequencing of their amplified DNA and comparison to known sequences by BLAST analysis against the NCBI database (Folmer et al. 1994). Arthropods preserved in ethanol were dried overnight at room temperature, and thoroughly washed using distilled water. Individual grasshoppers, crickets, cockroaches, and beetles were kept separate for screening, while red harvester ants and desert termites were pooled by species (≤ 20 specimens/pool) based on trapping locations. Screening of arthropods for the presence of *O.petrowi* was accomplished by extracting DNA from individual and pooled specimens using the DNeasy Blood & Tissue Kit (QIAGEN, Maryland, USA). Specimens were then analyzed for the presence of eyeworms using the *O. petrowi*-specific primers (OP3/OP4) designed to the ITS2 region, as described above. Primers were sensitive enough to detect 1 pg genomic DNA of *O. petrowi*. To ensure the reliability and validity of the PCR assay, negative and positive controls containing DNA of *Strongyloides stercoralis* and *O. petrowi* were run along with the samples, respectively. To further validate results, amplified PCR products from ten positive samples were cloned into JM109 competent cells according to the manufacturer’s (Promega Corp., Wisconsin, USA) instructions, then submitted for genetic sequencing to the University of Maine DNA Sequencing Facility (Orono, Maine, USA).

## Results

Primers SPIR 18F and 28S/408R/20 amplified the entire ITS region, including a partial 18S, ITS1, 5.8S, ITS2, and a partial 28S. The sequence was deposited into the NCBI database with accession number KF 306222 to make it publicly accessible. The designed primers to the ITS*2* region were very effective (100%) in amplifying the target region of *O. petrowi*.

Total arthropod samples included 6 beetles, 11 crickets, 22 katydids, 34 cockroaches, 289 grasshoppers, 34-pooled samples of desert termites, and 134 pooled samples of red harvester ants. Of the arthropods determined to be positive for *O. petrowi* by PCR, 38% (13/34) of the cockroaches, 27% (3/11) of the crickets, and 23% (68/289) of the grasshoppers were determined to contain DNA of *O. petrowi*. All of the red ants, beetles, katydids, and termites were determined to be negative for the eyeworm. Out of the 15 different species of grasshoppers screened for the presence of *O. petrowi*, six different species of grasshoppers including *Melanoplus differentialis*, *Melanoplus femurrubrum*, *Trimerotropis pallidipennis*, *Melanoplus ponderosus*, *Opeia obscura*, and *Hippiscus ocelote* were positive for *O. petrowi*. Other than these grasshopper species, Texas field cricket (*Gryllus texensi*s) and wood cockroach (*Parcoblatta* spp.) were also determined to contain DNA of eyeworm (Table 1). To validate our results, the DNA of ten different specimens that were PCR-positive were cloned and sent for sequencing. Complete identity of ITS2 region of adult nematode with ITS2 rDNA sequence of these larval samples not only confirmed our results but also demonstrated the similarity of the sequences of larvae and adults.

**Table 1:** *Oxyspirura petrowi* infection rates in various Blattodea and Orthoptera arthropod species collected from the Rolling Plains ecoregion of Texas and Oklahoma

Arthropod species screened	Common name	Percentage positive for *O. petrowi*
*Melanoplus differentialis* ^a^	Differential grasshopper	40% (14/35)
*Parcoblatta* spp*.*^b^	Wood cockroach	38% (13/34)
*Melanoplus femurrubrum* ^a^	Red-legged grasshopper	32% (10/31)
*Trimerotropis pallidipenni*s^a^	Pallid-winged grasshopper	32% (9/28)
*Melanoplus ponderosus* ^a^	Spur-throated grasshopper	31% (19/62)
*Opeia obscura* ^a^	Obscure grasshopper	31% (4/13)
*Gryllus texensis* ^c^	Texas field cricket	27% (3/11)
*Hippiscus ocelote* ^a^	Wrinkled grasshopper	16% (12/73)
*Syrbula montezuma* ^a^	Montezuma’s grasshopper	0% (0/1)
*Leprus wheeleri* ^a^	Wheeler’s blue-winged grasshopper	0% (0/1)
*Hesperotettix viridis pratensis* ^a^	Purple-striped grasshopper	0% (0/3)
*Boopedon nubilum* ^a^	Ebony grasshopper	0% (0/4)
*Boopedon gracile* ^a^	Graceful range grasshopper	0% (0/5)
*Hadrotettix trifasciatus* ^a^	Three-banded grasshopper	0% (0/5)
*Brachystola magna* ^a^	Plains lubber grasshopper	0% (0/8)
*Schistocerca obscura* ^a^	Obscure bird grasshopper	0% (0/10)
*Campylacantha olivacea* ^a^	Fuzzy olive-green grasshopper	0% (0/10)
*Pediodectes haldemani* ^d^	Haldeman’s shieldback katydid	0% (0/22)
*Pogonomyrmex barbatus* ^e^	Red harvester ants	0% (0/134) pooled samples
Gnathamitermes tubiformans^f^	Desert termites	0% (0/34) pooled samples
Unknown	Beetles	0% (0/6)

^a^Orthoptera: Acrididae

^b^Blattodea: Ectobiidae

^c^Orthoptera: Gryllidae

^d^Orthoptera: Tettigoniidae

^e^Hymenoptera: Formicidae

^f^Blattodea: Termitidae

## Discussion

The objective for this project was to develop a PCR-based method to identify potential intermediate arthropod hosts for *O. petrowi*. Our efforts consisted of amplifying the ITS1/5.8S/ITS2 region of ribosomal DNA (rDNA) of the nematode and designing primers to target its ITS2 region. To the best of our knowledge, there is no method reported for the screening of arthropods for the presence of *O. petrowi* other than the traditional method of visual inspection. The desired approach towards designing primers specific for *O. petrowi* would have included sequence alignment of all congeners that were sympatric, which in this case would have been *O. pusillae*, because *O. petrowi* and *O. pusillae* have been reported to occur in the same areas (Pence 1972). Further, it would have been beneficial to understand the level of intraspecific variation at this locus for this group of parasites, but the lack of sequence data for *O. pusillae* and *O. mansoni* in the NCBI database and lack of access to worms of these species for PCR made this unfeasible. However, even if the sequence obtained from an arthropod showed 100% identity with the sequence of *O. petrowi*, without the proper knowledge of the intraspecific variation within this genus, it is difficult to conclude that the insect was indeed infected with *O. petrowi* and not some other species of this genus that is prevalent in that locality. Arguments posed by Pence with respect to the occurrence of *O. petrowi* and *O. pusillae* in different ecological niches, as well as the identification of all adult worms collected in this study as *O. petrowi*, strongly suggest that *O. petrowi* is the only species of *Oxyspirura* prevalent in the study areas (Pence 1972).

To validate our primers, we compared the ITS1 region of *O. petrowi* with the ITS1 sequence of the only species of *Oxyspirura* present in the NCBI database, *O. conjunctivalis*. A comparison of the ITS2 regions were not possible because there are no sequence data for the ITS2 region of *O. conjunctivalis*, nor for any species taxonomically related to the eyeworms of the genus *Thelazia*, in the NCBI database. The ITS1 regions of the *Oxyspirura* species showed very few similarities, indicating the rapid evolution of the ITS region in spiruroid nematodes (Ivanova et al. 2007). Thus, because of the sufficient level of variation, this region was considered capable of being used to discriminate between species of the same genus. Additionally, the ITS region had been proven to be a reliable genetic marker for the identification of *Thelazia* spp. in arthropods serving as intermediate hosts (Traversa et al. 2005). Grasshoppers were abundantly available in the study areas and were the insect commonly found in the crops of quail, and they constituted the major portion of the arthropod specimens collected (Jackson 1969).

Our findings identified different species of arthropods that may be serving as the intermediate host of *O. petrowi* in this ecoregion. Because these arthropods can harbor and transmit many other parasites, a molecular analysis based on the ITS2 region was performed to specifically identify *O. petrowi*. The presence of *O. petrowi* in a variety of insects illustrates its general behavior at the intermediate host level, and its potential to invade a variety of insects. This wide range of potential intermediate hosts and its numerous definitive hosts makes the survival of *O. petrowi* possible over a large geographical range.
